# An Alternative Approach to Managing Urethrovesical Anastomotic Leakage After Robot-Assisted Laparoscopic Radical Prostatectomy: External Urethral Meatus Catheter Fixation

**DOI:** 10.7759/cureus.82889

**Published:** 2025-04-24

**Authors:** Yushi Miyata, Hiroshi Kiuchi, Fumie Yoshioka, Tetsuji Soda, Kenichiro Sekii

**Affiliations:** 1 Urology, Osaka Central Hospital, Osaka, JPN

**Keywords:** anastomotic urinary leakage, catheter fixation, external urethral meatus, management, rarp, urinary peritonitis

## Abstract

A 46-year-old male presented with a prostate-specific antigen (PSA) level of 4.27 ng/mL. MRI identified a Prostate Imaging Reporting and Data System category 4 lesion in the right peripheral zone. Prostate biopsy confirmed adenocarcinoma with a Gleason score of 3+3. The patient was diagnosed with localized prostate cancer (cT2aN0M0) and underwent robot-assisted laparoscopic radical prostatectomy via a transperitoneal approach. Postoperatively, urine was immediately observed through the intraperitoneal drain. To address the anastomotic urinary leakage (AUL), gentle traction of the urethral catheter was applied and secured with abdominal tape. However, on postoperative day 8, the patient developed severe abdominal pain. A CT scan revealed fluid accumulation around the bladder, liver, and spleen, along with ventral migration of the drain, indicating inadequate urine drainage. Standard AUL management strategies had proven ineffective, complicating the case. Ultimately, fixation of the urethral catheter at the external urethral meatus using adhesive tape successfully resolved the leakage and alleviated the abdominal pain. This simple technique may offer an effective alternative for managing AUL.

## Introduction

The overall complications following robot-assisted laparoscopic radical prostatectomy (RARP) occur in approximately 10% of cases [1,2]. Among these, anastomotic urinary leakage (AUL) is a relatively common complication, with an incidence ranging from 0.1% to 6.7% [2]. In most instances, AUL can be managed with simple interventions such as gentle traction of the urethral catheter secured with abdominal tape or prolonged catheterization, and serious clinical consequences are uncommon [1,3]. However, in our case, conventional AUL management proved ineffective, resulting in urine accumulation within the peritoneal cavity and severe abdominal pain. Despite the failure of standard treatments, a straightforward method - securing the urethral catheter at the external urethral meatus with adhesive tape - successfully resolved the leakage and relieved the abdominal pain. Here, we present this case along with a review of the relevant literature.

## Case presentation

**Figure 1:**
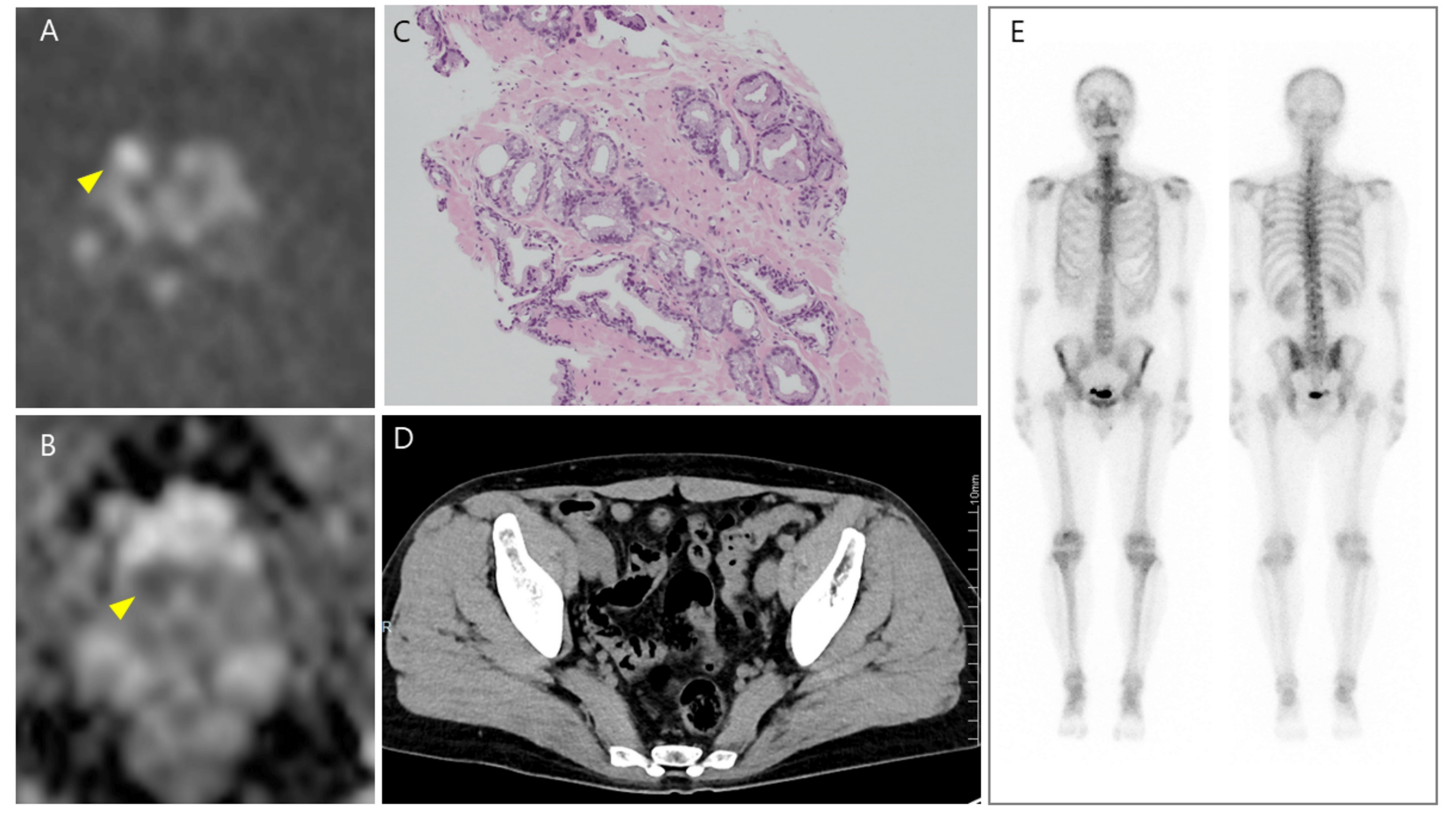
(A, B) MRI revealed a Prostate Imaging Reporting and Data System category 4 lesion in the right peripheral zone (arrowhead). (C) Histopathological analysis confirmed a Gleason score of 3+3 adenocarcinoma. (D) CT and (E) bone scintigraphy showed no distant metastases.

A 46-year-old male presented with an elevated prostate-specific antigen level of 4.27 ng/mL (reference range: <4.0 ng/mL) during a health check and was referred to our department. MRI revealed a Prostate Imaging Reporting and Data System 4 lesion in the right peripheral zone, prompting a prostate biopsy (Figure 1A, 1B). Histopathological analysis confirmed a Gleason score of 3+3 adenocarcinoma in 3 of 13 biopsy cores (Figure 1C). Bone scintigraphy and thoracoabdominal CT showed no distant metastases, confirming localized prostate cancer (cT2aN0M0) (Figure 1D, 1E). The patient underwent RARP via a transperitoneal approach. During surgery, partial injury to the posterior bladder wall occurred during bladder neck dissection, which was repaired with 3-0 Vicryl™ sutures. Urethrovesical anastomosis was performed in two layers using barbed sutures (3-0 V-Loc™), reinforced with a Rocco stitch. Intraoperative bladder irrigation with saline revealed leakage from the dorsal anastomotic site, necessitating re-anastomosis. A Foley catheter (18 Fr, cuff 10 mL) was placed, and the procedure was completed.

**Figure 2:**
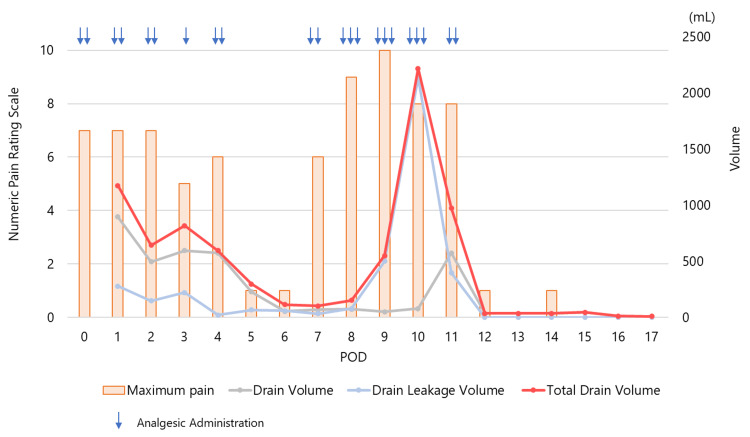
Correlation between the Numeric Pain Rating Scale and total drain output, including both drain volume and leakage volume The Numeric Pain Rating Scale is an 11-point scale ranging from 0, indicating no pain, to 10, indicating the worst pain imaginable.

**Figure 3:**
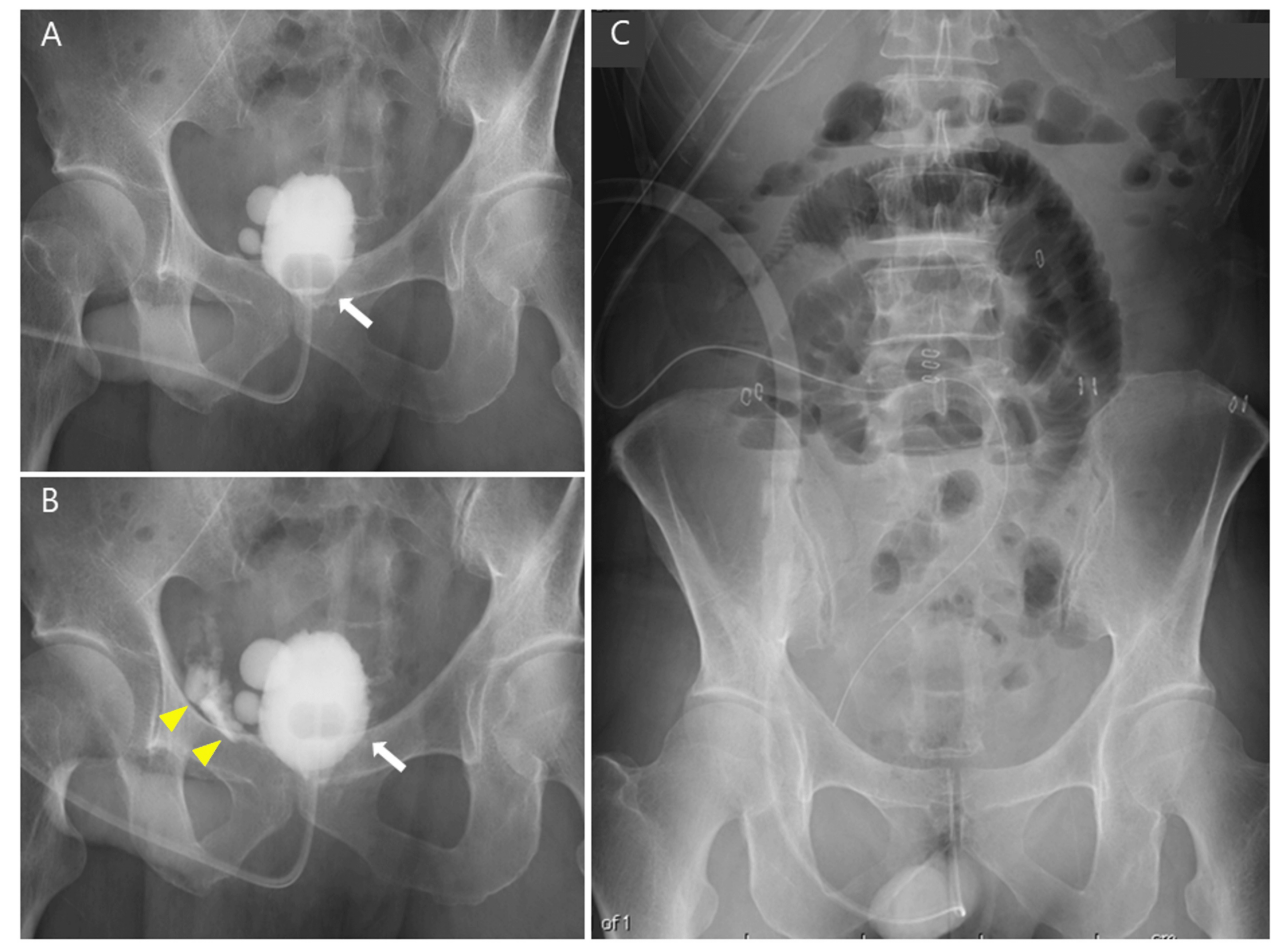
(A) Cystography on POD 2 showing no contrast leakage when gentle traction was applied to the catheter balloon at the anastomotic site. The arrow indicates the location of the catheter balloon. (B) Upon release of the traction, contrast leakage was observed at the 6 o’clock position. The arrowhead indicates the leakage site. (C) Abdominal X-ray on POD 4 demonstrating signs of ileus. POD, postoperative day

Postoperatively, significant drainage output was noted from the intraperitoneal drain, along with leakage around the drain, resulting in a total volume of 1174 mL/day on postoperative day (POD) 1 (Figure 2). On POD 2, due to persistent high drainage output, a cystography was performed. No contrast leakage was observed when the catheter balloon was gently pulled against the anastomotic site. However, upon releasing the traction, contrast leakage appeared around the 6 o’clock position, confirming AUL (Figure 3A, 3B). The urethral Foley catheter was replaced with a renal pelvis balloon catheter (14 Fr, cuff 5 mL) to facilitate efficient bladder drainage. Oral intake was initiated on POD 1, but the patient developed nausea and sudden severe abdominal pain on POD 4. Abdominal radiography revealed findings suggestive of ileus, leading to a diagnosis of ileus-related pain (Figure 3C), which improved within a few days following bowel rest. Meanwhile, the persistently high drainage output (597 mL/day) on POD 4 indicated ongoing AUL. Gentle traction was applied to the urethral catheter, and it was secured to the abdominal wall. This management led to a gradual reduction in drainage output and pain relief by POD 6 (116 mL/day). 

**Figure 4:**
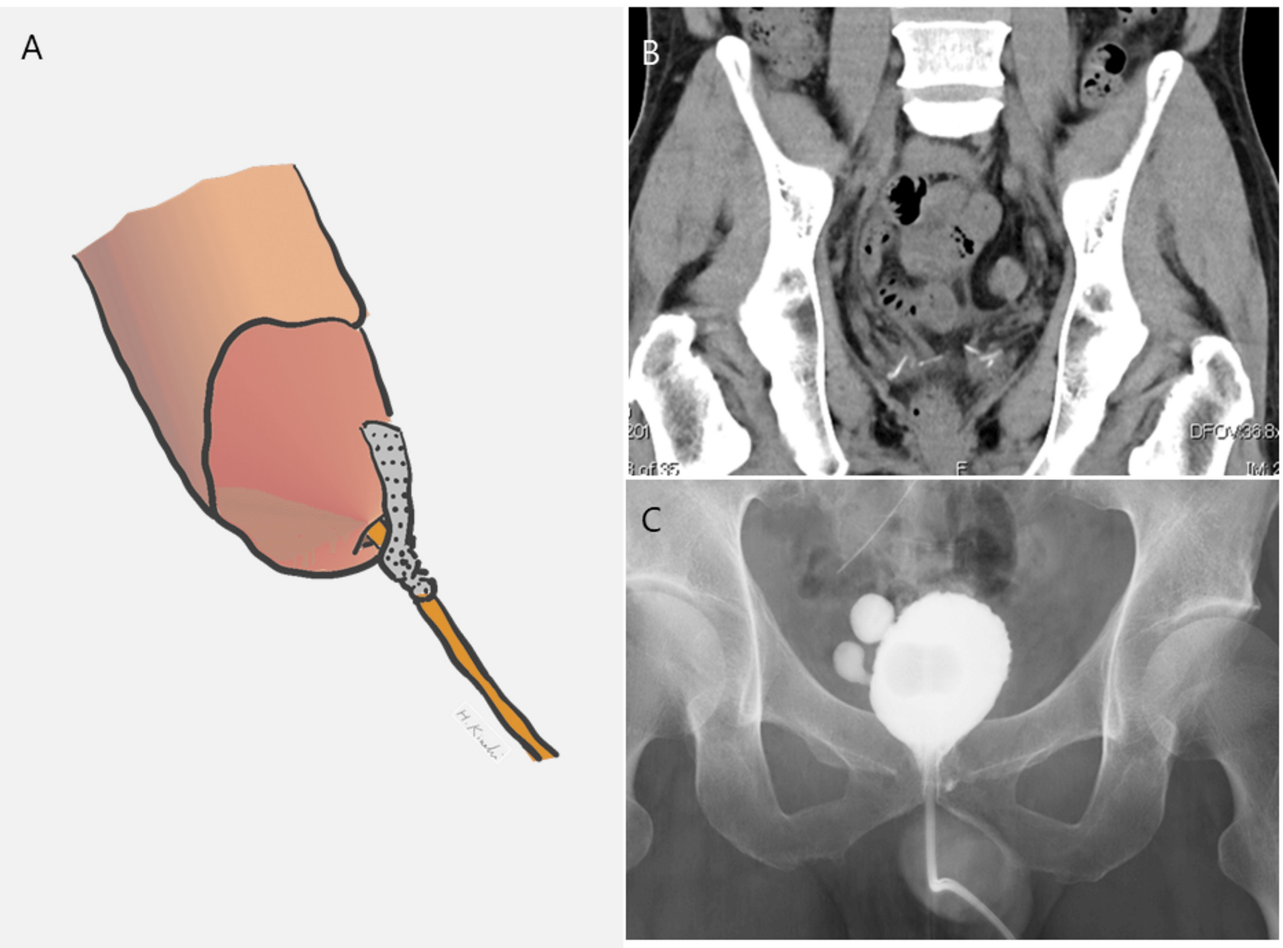
(A) A novel and simple technique for managing AUL: schematic illustration of urethral catheter fixation at the external urethral meatus using adhesive tape (original schema). (B) Abdominal CT scan on POD 17 showing no fluid accumulation. (C) Cystography on POD 21 revealing only minor residual leakage. AUL, anastomotic urinary leakage; POD, postoperative day

However, on the night of POD 8, severe abdominal pain recurred, followed a few days later by a sharp increase in drain leakage output, which peaked at 2,218 mL/day on POD 10, accompanied by intolerable pain. CT imaging revealed fluid accumulation around the bladder, liver, and spleen, with ventral migration of the drain, impairing urinary drainage (Figure 4A-4C). Ileus was not observed on CT. Blood tests showed a transient elevation in BUN, creatinine, and potassium (Table 1). Given the increasing drain output, worsening pain, and intraperitoneal fluid accumulation secondary to AUL, a state of peritoneal autodialysis was indicated. Antibiotic therapy with flomoxef sodium was initiated, and severe pain was managed by increasing the frequency of analgesic administration.

**Table 1:** Perioperative changes in serum BUN, creatinine, potassium levels, WBC, and CRP The asterisk (*) indicates a transient elevation. POD, postoperative day; RARP, robot-assisted laparoscopic radical prostatectomy

Parameter	Reference range	Pre-RARP	POD4	POD6	POD10	POD12	POD17
BUN, mg/dL	8-21	7	11	10	21*	11	9
Cr, mg/dL	0.0-1.0	0.81	0.65	0.56	1.52*	0.57	0.59
K, mEq/L	3.5-5.3	3.5	4.0	4.3	4.7*	4.4	4.1
WBC, /mm³	3,200-8,500	7,800	15,740	6,230	9,320*	7,760	7,020
CRP, mg/dL	0.0-0.40	0.03	4.24	2.71	1.05	1.18	0.30

**Figure 5:**
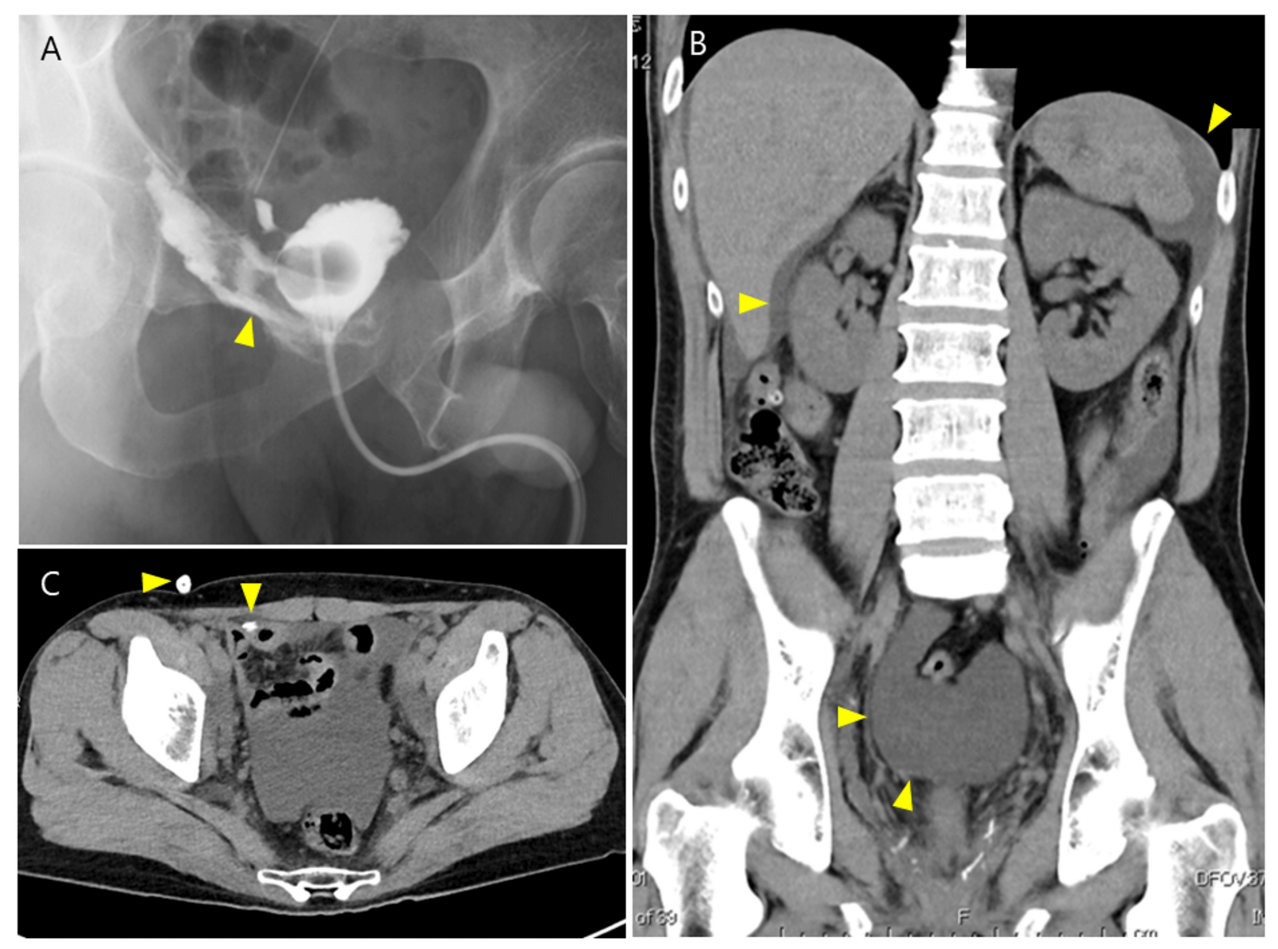
(A) Cystography on POD 8 demonstrating significant enlargement of the anastomotic leakage. The arrowhead indicates contrast leakage. (B) Abdominal CT scan on POD 9 showing fluid accumulation around the bladder, liver, and spleen. (C) Ventral migration of the drain, impairing urinary drainage. The arrowhead indicates the position of the drain. POD, postoperative day

Urine output was higher during ambulation but decreased when the patient lay down in bed. Based on this observation, we hypothesized that lying down caused the balloon to shift away from the bladder neck, leading to AUL recurrence. To maintain the balloon’s position at the bladder neck even in a supine position, the urethral catheter was secured at the external urethral meatus using adhesive tape instead of abdominal fixation (Figure 5A). Following this intervention, drainage output rapidly decreased to 37 mL/day by POD 12, and pain subsided. CT on POD 17 confirmed resolution of intraperitoneal fluid accumulation (Figure 5B). Cystography on POD 21 showed only minor residual leakage (Figure 5C), allowing for catheter removal and hospital discharge on POD 25. 

## Discussion

AUL is a well-recognized complication of RARP, with an incidence ranging from 0.1% to 6.7% [1,4,5]. Notably, this complication occurs less frequently in RARP compared to open radical prostatectomy, where the incidence is 3.5-10.0%. In most cases, AUL resolves with simple interventions such as urethral catheter traction and/or prolonged catheterization [3]. However, significant urine leakage with inadequate drainage can result in substantial intraperitoneal urine accumulation, potentially leading to severe complications such as pain, urinary peritonitis, ileus, renal failure, and sepsis [3,6].

In our case, abdominal pain persisted from the immediate postoperative period, initially attributed to postoperative wound pain or ileus. However, the presence of intraperitoneal fluid retention was confirmed on CT at POD 10. As shown in Figure 1, the intensity of maximum pain was greater during periods of increased drain output and alleviated as the drain output decreased. This suggests that the persistent pain and ileus may have been, in part, due to intraperitoneal urine accumulation.

Since most cases of AUL following RARP involve only minor leakage at the anastomotic site, standard management and/or appropriate urinary drainage are typically sufficient to prevent severe complications. In this case, urine drained both through and around the drain, leading to the assumption that drainage was adequate. However, CT at POD 10 revealed fluid accumulation around the liver and spleen, indicating inadequate drainage. These findings highlight the importance of vigilance when a large volume of urine is drained through the drain or extraluminal pathways, as inadequate drainage may go unnoticed. Additionally, elevated levels of serum BUN, creatinine, and potassium serve as useful diagnostic indicators for intraperitoneal urine accumulation. This condition, known as pseudo-renal failure, occurs due to peritoneal autodialysis [7], which was observed in our case.

Management of intraperitoneal urine accumulation due to AUL following RARP involves drainage of both intraperitoneal and bladder urine, along with, if necessary, administration of broad-spectrum antibiotics [8,9], and appropriate management for AUL. AUL following RARP is often suspected by increased postoperative drain output, and cystography is valuable for identifying the location and extent of the leakage.

Initial treatment typically involves gentle traction of the urethral catheter and securing it to the abdominal wall or thigh with tape (Table 2). This helps seal the damaged area with the catheter balloon, reducing urine leakage from the anastomosis and promoting wound healing. Excessive traction should be avoided, as it may induce ischemia at the anastomotic site or cause the balloon to dislocate downward from the bladder neck [10]. Conversely, if traction exacerbates anastomotic dehiscence, the balloon should be inserted deeper and secured with tape. Adjusting the balloon size, whether larger or smaller, can also improve the sealing of the anastomosis. Mochtar et al. suggested that fluoroscopic-guided catheter adjustments could improve treatment outcomes [11]. While the typical duration for catheter indwelling after RARP is three to six days, extending this period may enhance wound healing. When AUL persists despite minimal interventions, bilateral ureteral stenting is an alternative to prevent direct urine exposure to the anastomotic defect, facilitating wound healing. If conventional methods fail, alternative approaches must be considered. Bhatt et al. reported successful management by replacing the Foley catheter with a 16Fr pigtail catheter [12]. Additionally, Diamand et al. demonstrated that creating a proximal hole in the Foley catheter balloon effectively resolved AUL [13].

**Table 2:** Management of AUL and its level of invasiveness for the patient AUL, anastomotic urinary leakage

Management of AUL	Level of invasiveness for the patient
Gentle traction of the urethral catheter with fixation (abdominal wall, thigh, or external urethral meatus)	Minimal
Adjustment of the cuff location	Minimal
Adjustment of the catheter cuff volume (increase or decrease)	Minimal
Prolonged urethral catheterization	Minimal
Bilateral ureteral stent placement	Moderate
Re-suturing of the anastomosis	Severe

In our case, cystography did not clearly indicate whether the AUL originated from the anastomotic site or a posterior bladder wall injury. Based on the cystographic findings, we applied gentle traction to the catheter and secured it to the abdominal wall with tape. Although this initially reduced the drain output, the volume subsequently increased, accompanied by abdominal pain. Urine output was higher during ambulation but decreased when the patient lay down. Based on this observation, we hypothesized that lying down caused the balloon to shift away from the bladder neck, leading to AUL recurrence. To address this, we fixed the catheter at the external urethral orifice to maintain balloon stability regardless of positional changes. This adjustment resulted in a rapid decrease in drain output and eventual resolution of the AUL. This simple method of securing the catheter at the external urethral orifice was effective in improving AUL. To prevent serious complications, such as urinary peritonitis following AUL, a pelvic approach during RARP or peritoneal closure during transperitoneal RARP may be considered. However, in most cases, AUL can be managed with conventional methods. When these methods are ineffective, familiarity with alternative treatment approaches is essential.

## Conclusions

AUL following RARP typically resolves with standard management techniques, such as gentle traction of the urethral catheter or prolonged catheterization, with clinically significant complications being rare. However, when conventional management fails, AUL can lead to severe complications. Fixating the urethral catheter at the external urethral meatus using adhesive tape offers a simple and effective solution to resolve AUL. This technique is a feasible and practical treatment option in the management of AUL.
